# Optimizing left ventricular-arterial coupling during the initial resuscitation in septic shock – a pilot prospective randomized study

**DOI:** 10.1186/s12871-021-01553-w

**Published:** 2022-01-21

**Authors:** Xiaoyang Zhou, Yiqin Zhang, Jianneng Pan, Yang Wang, Hua Wang, Zhaojun Xu, Bixin Chen, Caibao Hu

**Affiliations:** 1 Department of Intensive Care Medicine, HwaMei Hospital, University of Chinese Academy of Sciences, Ningbo, 315000 Zhejiang China; 2 Ningbo Institute of Life and Health Industry, University of Chinese Academy of Sciences, Ningbo, 315000 Zhejiang China; 3 Department of Emergency, Ningbo Yinzhou No.2 Hospital, Zhejiang 315000 Ningbo, China; 4 Department of Intensive Care Medicine, Affiliated Zhejiang Hospital, Zhejiang University School of Medicine, Hangzhou, 310000 Zhejiang China

**Keywords:** Ventricular-arterial coupling, Cardiac stroke work, Septic shock, Resuscitation, Prognosis

## Abstract

**Background:**

Left ventricular-arterial coupling (VAC), defined as the ratio of effective arterial elastance (Ea) to left ventricular end-systolic elastance (Ees), has been extensively described as a key determinant of cardiovascular work efficacy. Previous studies indicated that left ventricular-arterial uncoupling was associated with worse tissue perfusion and increased mortality in shock patients. Therefore, this study aims to investigate whether a resuscitation algorithm based on optimizing left VAC during the initial resuscitation can improve prognosis in patients with septic shock.

**Methods:**

This pilot study was conducted in an intensive care unit (ICU) of a tertiary teaching hospital in China. A total of 83 septic shock patients with left ventricular-arterial uncoupling (i.e., the Ea/Ees ratio ≥ 1.36) were randomly assigned to receive usual care (usual care group, *n* = 42) or an algorithm-based resuscitation that attempt to reduce the Ea/Ees ratio to 1 within the first 6 h after randomization (VAC-optimized group, *n* = 41). The left VAC was evaluated by transthoracic echocardiography every 2 h during the study period. The primary endpoint was 28-days mortality. The secondary endpoints included lactate clearance rate, length of ICU stay, and duration of invasive mechanical ventilation (IMV).

**Results:**

Eighty-two patients (98.8%) completed the study and were included in the final analysis. The Ea/Ees ratio was reduced in both groups, and the decrease in Ea/Ees ratio in the VAC-optimized group was significantly greater than that in the usual care group [median (interquartile range), 0.39 (0.26, 0.45) vs. 0.1 (0.06, 0.22); *P* < 0.001]. Compared with the usual care group, the VAC-optimized group likely exhibited the potential to reduce the 28-days mortality (33% vs. 50%; log-rank hazard ratio = 0.526, 95% confidence interval: 0.268 to 1.033). Moreover, the VAC-optimized group had a higher lactate clearance rate than the usual care group [27.7 (11.9, 45.7) % vs. 18.3 (− 5.7, 32.1) %; *P* = 0.038]. No significant difference was observed in terms of the length of ICU stay or duration of IMV.

**Conclusions:**

During the initial resuscitation of septic shock, optimizing left ventricular-arterial coupling was associated with improved lactate clearance, while likely having a beneficial effect on prognosis.

**Trial registration:**

Chinese Clinical Trial Registry, ChiCTR1900024031. Registered 23 June 2019 - Retrospectively registered.

**Supplementary Information:**

The online version contains supplementary material available at 10.1186/s12871-021-01553-w.

## Background

In the circulatory system, the cardiac ventricle works together with the arterial system in an interdependent and interactive manner, which refers to left ventricular-arterial coupling (VAC) [1]. Left VAC is calculated as the ratio of effective arterial elastance (Ea) to left ventricular end-systolic elastance (Ees) [2, 3]; an Ea/Ees ratio greater than 1.36 was considered as left ventricular-arterial uncoupling [4, 5].

From the perspective of myocardial energy metabolism, ventricular ejection is, in reality, a process where the left ventricle overcomes afterload and performs external work [6], and cardiac stroke work is the largest part of total cardiac mechanical energy [7]. A huge body of evidence suggested that the maximal cardiac stroke work occurred when the cardiovascular interaction was coupled (i.e., the Ea/Ees ratio = 1) [6, 8, 9]. Moreover, increasing cardiac stroke work was significantly related to improved tissue perfusion [10] and the improvement of VAC during the resuscitation from shock was associated with improved stroke work and systemic tissue perfusion [11]. Thus, it is reasonable to assume that optimizing left VAC during the early resuscitation could improve prognosis in patients with septic shock who usually has mismatched ventricular-arterial interactions [5, 12].

Early goal-directed therapy (EGDT) represents a landmark resuscitation strategy in the treatment of septic shock. However, recent studies [13–15] failed to identify any survival benefit with the implementation of EGDT in the resuscitation of septic shock. A potential explanation for the negative effect may be that EGDT defines homogeneous hemodynamic targets for all septic shock patients who, however, generally have a complex and heterogeneous cardiovascular profile. For instance, an initial target mean arterial pressure (MAP) of 65 mmHg seems to be insufficient for septic shock patients with prior hypertension. Since EGDT recommends multiple treatment measures (fluid expansion, inotropes, vasoactive drugs, etc.) that have complex effects on cardiovascular performance [16–19], homogeneous hemodynamic targets provided by EGDT might result in cardiovascular nonequilibrium (namely left ventricular-arterial uncoupling), which was associated with worse outcomes in septic shock patients [12]. In this regard, individualized treatment adjustment based on optimizing the cardiovascular interaction may be a promising supplement to EGDT strategy, and dynamically monitoring left VAC may facilitate better guiding the adjustment of treatment during the initial resuscitation.

In this study, we formulated a resuscitation algorithm that aimed to optimize the left VAC to 1 and verified its applicability in patients with septic shock. More importantly, we tested the hypothesis that optimizing left VAC during the initial resuscitation from septic shock can improve prognosis.

## Materials and methods

This prospective open-label study was performed in the intensive care unit (ICU) of HwaMei Hospital, University of Chinese Academy of Sciences, a tertiary teaching hospital in China, between January 2019 and January 2021. This study was approved by the local institutional ethics committee (PJ-NBEY -KY-2019-014-01). All methods were performed following the CONSORT guidelines. Written informed consent was obtained from patients or their relatives. The study protocol was registered at the Chinese Clinical Trial Registry (ChiCTR1900024031).

### Study participants

Adult patients with septic shock (age > 18 years) were screened for enrollment after their ICU admission. The diagnosis of septic shock was based on the third international consensus definition for sepsis and septic shock [20]. Those patients who had uncoupled left ventricular-arterial interactions (i.e., the Ea/Ees ratio ≥ 1.36) at the time of enrollment were eligible. The exclusion criteria included: 1) patients with an Ea/Ees ratio < 1.36 at enrollment; 2) patients with atrial fibrillation; 3) patients who underwent cardiac surgery; 4) patients with cardiac function assist device (such as a pacemaker); 5) patients with contraindications to inotropes; 6) patients who had poor echogenicity or could not tolerate transthoracic echocardiography (TTE) examination; 7) refractory shock patients who were expected to die within 24 h after enrollment; 8) patients who declined to participate.

Eligible patients were randomly allocated to receive usual care (usual care group) or an algorithm-based resuscitation that attempts to reduce the Ea/Ees ratio to 1 within the first 6 h after randomization (VAC-optimized group) in a 1:1 ratio by using a computer-generated randomization method (SPSS 17.0, IBM, New York, USA). Due to the requirement to dynamically adjust the treatment during the resuscitation, the physicians in charge were not blinded to the random allocation.

### Study protocol

**Fig. 1:**
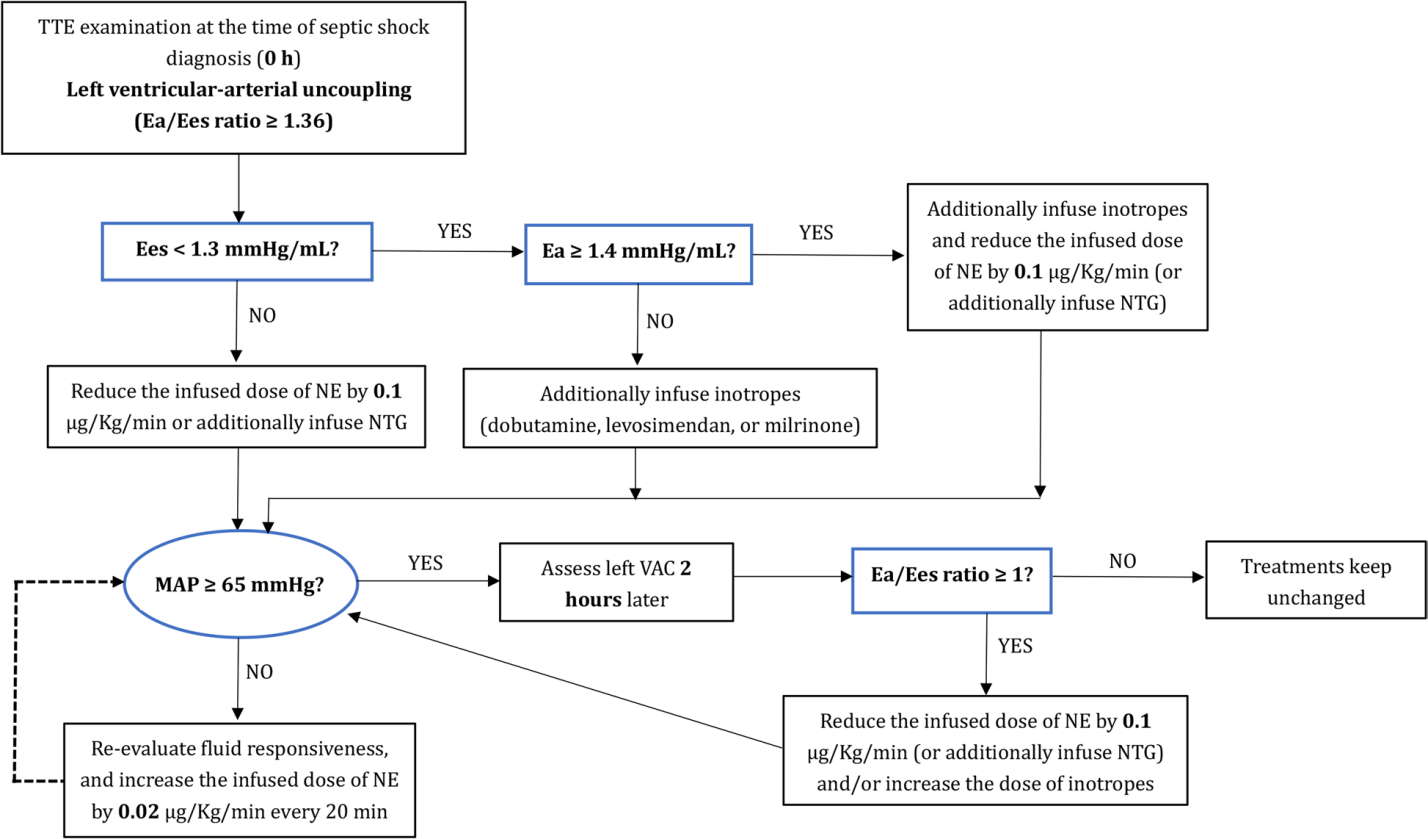
Resuscitation algorithm based on optimizing the left ventricular-arterial coupling. TTE transthoracic echocardiography; VAC ventricular-arterial coupling; Ea effective arterial elastance; Ees left ventricular effective end-systolic elastance; NE norepinephrine; NTG nitroglycerin

After admitting to the ICU, all eligible patients were continuously monitored with electrocardiogram, pulse oxygen saturation, and central venous pressure (CVP). Radial artery catheterization was inserted into all patients to measure invasive arterial blood pressure. During the initial resuscitation, the treatment strategy adhered to the recommendation of the international guidelines [21] and its update [22]. The treatment measures included fluid expansion, inotropes, vasoactive medications, blood transfusion, appropriate antibiotic therapy, infection source control, and organ support. Before giving more fluids, we assessed preload dependency by using dynamic echocardiographic indices, such as the respiratory variation in inferior vena cava diameter or the SV variation induced by passive leg raising or fluid challenge. Pressure-controlled ventilation mode was applied for all patients treated with invasive mechanical ventilation (IMV); sedative and analgesic drugs were administered to facilitate ventilation. During the study period, if not necessary, modifications of ventilator parameters or the dose of sedative and analgesic drugs were not allowed. We formulated a resuscitation algorithm that attempts to optimize the left VAC to 1. The detailed resuscitation algorithm is shown in Fig. 1.

In the usual care group, whether adjust treatments was decided by the attending physician according to the recommended criteria of the international guidelines [21]. In the VAC-optimized group, adjustment of treatments was also based on the recommended criteria [21]. However, the adjustment of inotropes and vasoactive drugs adhered to the resuscitation algorithm (Fig. 1), and the goal was to reduce the Ea/Ees ratio to 1, providing that the target MAP (more than 65 mmHg) was guaranteed. As reported in the study by Chen et al. [4], the normal value of Ea and Ees in a healthy adult population without a history of cardiovascular diseases was 2.2 ± 0.8 mmHg/mL and 2.3 ± 1.0 mmHg/mL, respectively. Thus, an Ees less than 1.3 mmHg/mL was considered as an indication for using inotropes (including dobutamine, levosimendan, and milrinone) in our study. Notably, the type of inotrope and its infused dose at each adjustment was decided by the physicians in charge. When the measured Ea was greater than 1.4 mmHg/mL, we decided to reduce the infused dose of norepinephrine (NE) (by 0.1 μg/kg/min at each adjustment) because the increased Ea/Ees ratio might result from a relatively higher Ea value than Ees value. If necessary, infusion of nitroglycerin was allowed to reduce arterial load. In this study, fluid administration was not guided by the VAC-optimized algorithm because the uncertain effect of fluid expansion on the left VAC depends on fluid responsiveness [16, 23].

### Transthoracic echocardiography

TTE examination was performed for all patients by an independent, experienced ICU physician, who was blinded to the study protocol, using a Philips CX50 ultrasound system (Philips Medical System, Suresnes, France). To obtain a good cardiac ultrasound image, all patients were positioned in the left lateral decubitus position and simultaneously connected to an electrocardiogram when performing TEE. All measurements were performed every 2 h within the first 6 h after randomization, regardless of the respiratory cycle. The mean value of each variable was calculated by the average value of three consecutive measurements.

Left ventricular end-diastolic volume (LVEDV), left ventricular end-systolic volume (LVESV), and left ventricular ejection fraction (LVEF) were measured on an apical four-chamber view by using Simpson’s method. Aortic velocity-time integral (VTI), pre-ejection time (T_pre-e_), and total systolic time (T_tot-s_) were measured with continuous Doppler on an apical five-chamber view. The diameter of the left ventricular outflow tract (LVOT) was measured on a parasternal long-axis view. Simultaneously, we also recorded the heart rate (HR), systolic arterial pressure (SAP), diastolic arterial pressure (DAP), and MAP at the time of TTE examination. SV was calculated as VTI × LVOT area, and cardiac index (CI) was calculated as (SV × HR)/body surface area. Finally, Ea was estimated by the following formulation: Ea = (0.9 × SAP)/SV [24]. Ees was estimated by the noninvasive single-beat method proposed by Chen et al. [25]. This method assumed a load-independent linear ESPVR and a constant volume axis intercept of the relationship curve [17].

### Data collection and outcomes

The baseline characteristics included age, gender, body mass index, source of infection, and concomitant diseases. We recorded the acute physiology and chronic health evaluation (APACHE) II score and sequential organ failure assessment (SOFA) score at the time of enrolment. Ventilator settings, including tidal volume, driving pressure, and positive end-expiratory pressure (PEEP), were collected in detail for patients treated with IMV. Information regarding inotropes and sedative and analgesic drugs administered during the study period were also recorded. CVP was measured every 2 h during the study period. Arterial blood gas was measured at enrollment (0 h) and 6 h thereafter using the GEM Premier 3500 blood gas analyzer (Instrumentation Laboratory Company, Bedford MA, USA). We also recorded and analyzed the dose of NE administered, urine output per hour, and cumulative fluid volume before NE infusion and during the study period for all subjects. All patients were followed up to 28 days after randomization. The physicians who assessed outcomes were blinded to the patient assignment. The primary outcome was 28-days mortality, which was defined as death within 28 days after randomization regardless of the cause of death. The secondary outcomes were lactate clearance rate, length of ICU stay, and duration of IMV. The lactate clearance rate was calculated as (lactate level at 0 h - lactate level at 6 h)/ lactate level at 0 h × 100%.

### Statistical analysis

Sample size calculation was performed before initiating the study using the Power Analysis and Sample Size software. According to the findings of our previous study [26], septic shock patients with uncoupled ventricular-arterial interaction had a 28-day mortality of 56.8%, and patients with ventricular-arterial coupling had a 28-day mortality of 25%. To observe such a reduction in the 28-day mortality after optimizing the left VAC, we calculated that a sample size of 82 patients (41 per group) was required, with a power of 80%, a type I error of 5%, and a lost-to-follow-up rate of 10%.

All statistical analyses were performed using the statistical software SPSS 17.0 (IBM, New York, USA). The normality of the continuous data distribution was assessed using the Kolmogorov–Smirnov test. Continuous data are expressed as the mean ± SD or the median and interquartile range (IQR), as appropriate. Categorical variables were presented as frequency and percentages. Comparisons of continuous data between the usual care group and the VAC-optimized group were assessed using the Student’s t test if the data were distributed normally; otherwise, the Mann-Whitney U test was used. Intergroup comparisons of continuous variables between different time points were assessed using the Student’s paired t test or Mann-Whitney U test, as appropriate. The Chi-squared test or Fisher’s exact test was applied to categorical variables. The log-rank test was applied to compare the cumulative incidence of 28-days mortality between the two groups, and the Kaplan-Meier survival plot was constructed. The intra-observer reproducibility for these directly measured ultrasound variables (LVEDV, LVESV, VTI, T_pre-e_, and T_tot-s_) was evaluated by calculating the coefficient of variation and the least significant change from 10 randomly selected patients, and the results indicated an acceptable intra-observer reproducibility (see additional file 1: Table S1). A two-sided *P* value of less than 0.05 was considered to indicate statistical significance.

## Results

**Fig. 2:**
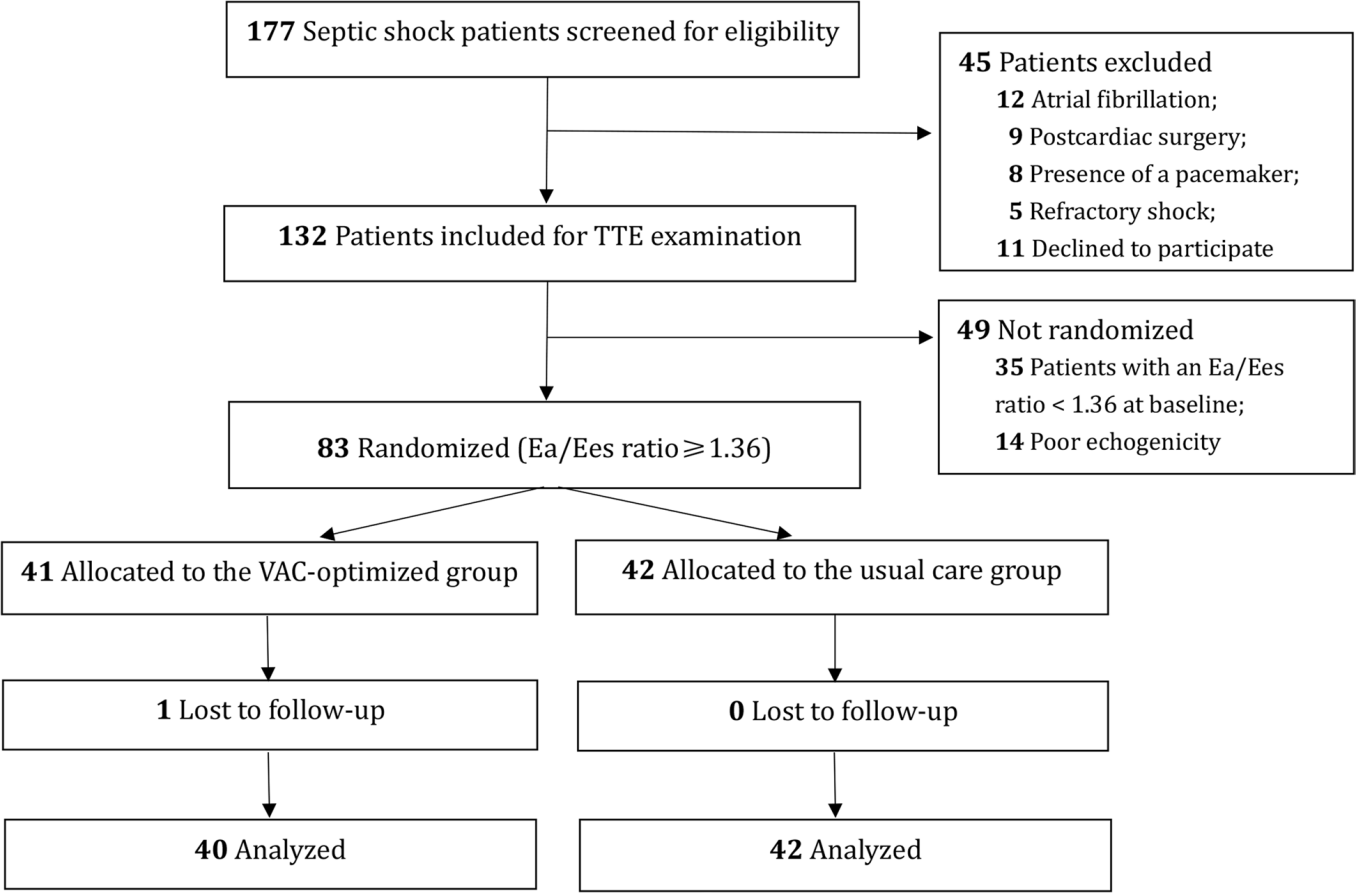
Flowchart of patient selection. TTE transthoracic echocardiography; Ea effective arterial elastance; Ees left ventricular effective end-systolic elastance; VAC ventricular-arterial coupling

Of the 177 septic shock patients admitted to the ICU from January 2019 to January 2021, 83 patients with left ventricular-arterial uncoupling underwent randomization and were assigned to either the usual care group (*n* = 42) or the VAC-optimized group (*n* = 41). As one patient in the VAC-optimized group was lost to follow-up, a total of 82 patients (98.8%) completed the study and were included in the final analysis (Fig. 2). As shown in Table 1, the usual care and VAC-optimized groups did not differ in terms of baseline characteristics. However, the usual care group had a higher incidence of bloodstream infection than the VAC-optimized group (*P* = 0.029).

**Table 1:** Baseline characteristics

Variables	Usual care group (*n* = 42)	VAC-optimized group (*n* = 40)
Age (years), mean ± SD	70 ± 12	68 ± 16
Male/Female, n	24/18	22/18
Body mass index (kg/m^2^), mean ± SD	22.9 ± 3.8	22.8 ± 3.4
APACHE II score at enrollment, mean ± SD	20 ± 5	21 ± 6
SOFA score at enrollment, mean ± SD	9 ± 3	9 ± 4
Time from ICU admission to randomization (hours), median (IQR)	2.0 (0.5, 8.5)	2.0 (1.0, 10.8)
Comorbidities, n (%)		
Hypertension	14 (33)	19 (48)
Diabetes	12 (29)	8 (20)
Chronic obstructive pulmonary disease	6 (14)	10 (25)
Chronic kidney disease	4 (10)	5 (13)
Coronary heart disease	3 (7)	4 (10)
Source of infection, n (%)		
Lung	21 (50)	23 (58)
Abdomen	10 (24)	8 (20)
Digestive tract	9 (21)	6 (15)
Bloodstream	9 (21)	2 (5)*
Urinary tract	2 (5)	7 (18)
Others	4 (10)	2 (5)
Invasive mechanical ventilation, n (%)	27 (64)	28 (70)
Ventilator settings, mean ± SD		
Tidal volume (mL/kg of PBW)	8.3 ± 2.0	8.2 ± 2.3
Driving pressure (cmH_2_O)	14 ± 4	14 ± 3
PEEP (cmH_2_O)	6 ± 2	7 ± 3
Analgesic and sedative drugs, n (%)		
Fentanyl	18 (43)	16 (40)
Midazolam	20 (48)	18 (45)
Propofol	7 (17)	10 (25)
Cumulative fluid volume before NE infusion (mL/kg), mean ± SD	19.0 ± 10.3	18.8 ± 10.4

* *P* value < 0.05 for the comparison between both groups

*VAC* ventricular-arterial coupling, *APACHE* acute physiology and chronic health evaluation, *SOFA* sequential organ failure assessment, *PBW* predicted body weight, *PEEP* positive end-expiratory pressure, *NE* norepinephrine, *IQR* interquartile range, *SD* standard deviation

**Fig. 3:**
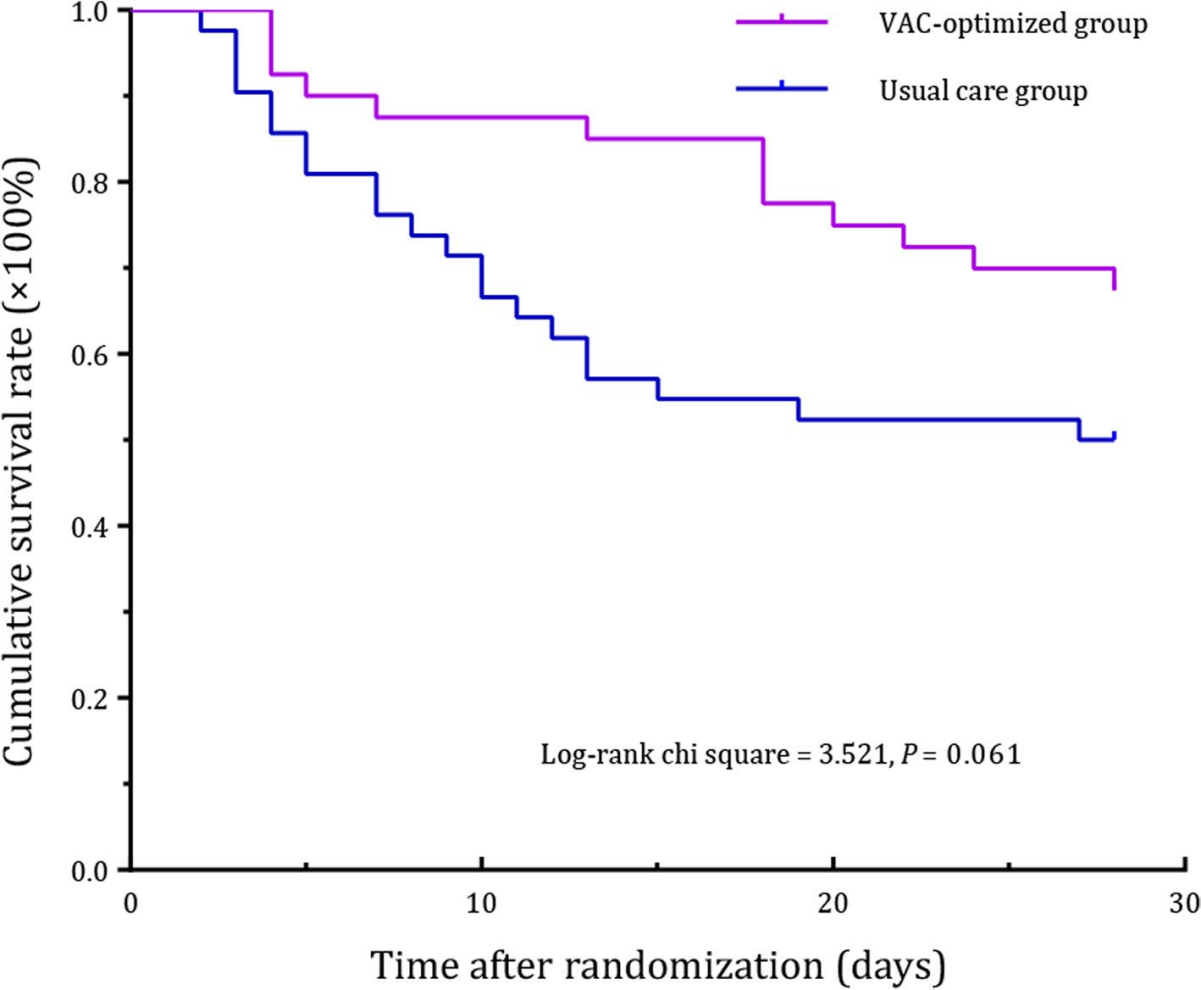
Kaplan-Meier survival plot. VAC ventricular-arterial coupling

As presented in Table 2, 21 patients (50%) in the usual care group and 13 patients (33%) in the VAC-optimized group died within 28 days after randomization. Optimizing the left ventricular-arterial coupling was likely associated with a reduced 28-days mortality (Log-rank hazard ratio = 0.526, 95% confidence interval: 0.268 to 1.033) (Fig. 3). Compared to the usual care, optimizing the left ventricular-arterial coupling improved the lactate clearance rate (*P* = 0.038), but did not affect the length of ICU stay or the duration of IMV. Notably, the proportion of patients who received inotropes in the VAC-optimized group was significantly higher than that in the usual care group (*P* = 0.025). In addition, optimizing the left ventricular-arterial coupling likely reduced the dose of norepinephrine administered (*P* = 0.094). However, the cumulative fluid volume during the study period and urine output in the VAC-optimized group did not differ from that in the usual care group. The dose of NE and inotropes at each time point were recorded detailly in Table S2 (additional file 1).

**Table 2:** Comparisons of clinical outcomes

Outcomes	Usual care group (n = 42)	VAC-optimized group (n = 40)	*P* value
Primary outcome			
28-day mortality, n (%)	21 (50)	13 (33)	0.061
Secondary outcomes			
Lactate clearance rate (%), median (IQR)	18.3 (−5.7, 32.1)	27.7 (11.9, 45.7)	0.038
Length of ICU stay (days), median (IQR)	9 (5, 13)	13 (5, 24)	0.106
Duration of IMV (days), median (IQR)	9 (4, 15)	9 (5, 23)	0.594
Other outcomes			
Dose of norepinephrine (μg/kg/min), median (IQR)	0.222 (0.148, 0.445)	0.196 (0.094, 0.301)	0.094
Inotropic drugs, n (%)	10 (24)	19 (48)	0.025
Dobutamine	10 (24)	17 (43)	0.072
Infused dose (μg/kg/min), mean ± SD	6.54 ± 2.63	5.49 ± 2.86	0.354
Levosimendan	0 (0)	1 (3)	0.488
Milrinone	0 (0)	2 (5)	0.235
Cumulative fluid volume during the study period (mL/kg), mean ± SD	22.1 ± 9.0	18.9 ± 13.9	0.216
Urine output (mL/kg/h), median (IQR)	1.15 (0.75, 1.79)	1.17 (0.66, 1.71)	0.659

*VAC* ventricular-arterial coupling, *ICU* intensive care unit, *IMV* invasive mechanical ventilation, *IQR* interquartile range, *SD* standard deviation

**Fig. 4:**
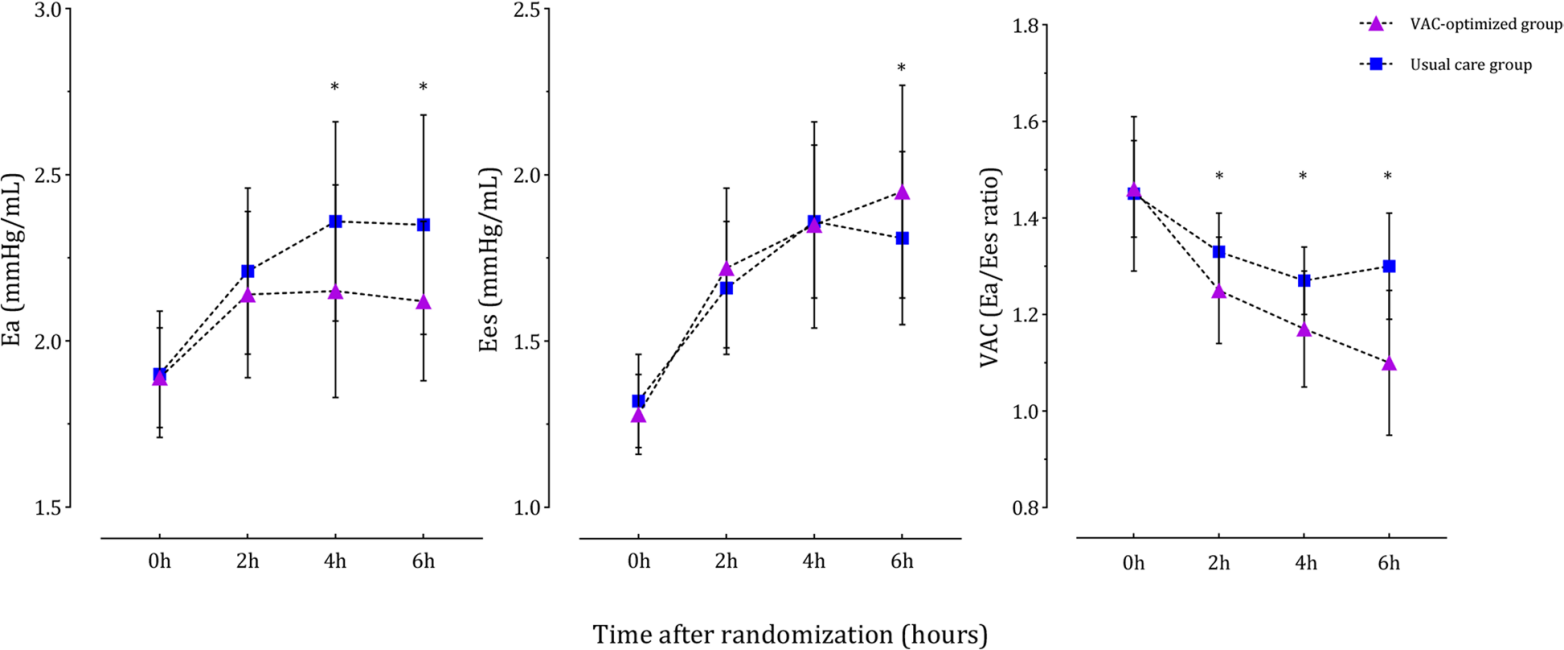
Comparisons of Ea, Ees, and VAC at each time point. * *P* < 0.05 for comparison between the usual care group and the VAC-optimized group. Ea effective arterial elastance; Ees left ventricular effective end-systolic elastance; VAC ventricular-arterial coupling

At baseline, the hemodynamic variables in the usual care group were comparable to the VAC-optimized group (Table 3). After 6 h of resuscitation, the arterial blood pressure was increased in both groups. The CVP was increased in the usual care group but unchanged in the VAC-optimized group. Additionally, the LVEDV and VTI were also increased in both groups, yet the LVESV was likely decreased. Thus, the LVEF was improved in both groups. Although the SV was increased after 6 h of resuscitation, the CI was decreased in both groups because of the reduced HR (Table 3). The decrease in Ea/Ees ratio in the VAC-optimized group was significantly greater than that in the usual care group [median (IQR), 0.39 (0.26, 0.45) vs. 0.1 (0.06, 0.22); *P* < 0.001]. At the end of the initial resuscitation, the VAC-optimized group had a higher Ees value, a lower Ea value, and a more coupled ventricular-arterial interaction than the usual care group. Comparisons of Ea, Ees, and Ea/Ees ratio between the two groups at each time point were illustrated in Fig. 4. The detailed changes of each variable at each time point were recorded in Table S3 (additional file 1).

**Table 3:** Changes of hemodynamics during the initial resuscitation

Hemodynamic variables	Usual care group (n = 42)		VAC-optimized group (n = 40)		*P* value ^a^	*P* value ^b^
	0 h	6 h	0 h	6 h		
HR (beats/min)	109 ± 19	90 ± 11*	113 ± 20	94 ± 10*	0.328	0.104
CVP (mmHg)	8 ± 3	10 ± 2*	9 ± 3	9 ± 2	0.206	0.016
SAP (mmHg)	92 ± 6	124 ± 11*	90 ± 5	114 ± 10*	0.118	< 0.001
DAP (mmHg)	44 ± 6	63 ± 7*	43 ± 4	63 ± 7*	0.364	0.949
MAP (mmHg)	60 ± 5	83 ± 8*	58 ± 4	80 ± 7*	0.175	0.063
SV (mL)	44 ± 6	48 ± 7*	44 ± 4	49 ± 5*	0.832	0.445
LVEDV (mL)	93 ± 11	96 ± 11*	96 ± 12	98 ± 11*	0.323	0.449
LVESV (mL)	49 ± 7	48 ± 5	50 ± 9	49 ± 7*	0.288	0.505
LVEF (%)	48 ± 4	50 ± 3*	46 ± 5	50 ± 4*	0.161	0.806
Cardiac index (L/min/m^2^)	2.9 ± 0.7	2.6 ± 0.4*	3.1 ± 0.6	2.9 ± 0.4*	0.216	0.006
Ea (mmHg/mL)	1.90 ± 0.19	2.35 ± 0.33*	1.89 ± 0.15	2.12 ± 0.24*	0.320	< 0.001
Ees (mmHg/mL)	1.32 ± 0.14	1.81 ± 0.26*	1.28 ± 0.12	1.95 ± 0.32*	0.244	0.037
Ea/Ees ratio	1.45 ± 0.16	1.30 ± 0.11*	1.46 ± 0.10	1.10 ± 0.15*	0.915	< 0.001
Lactate level (mmol/L)	3.1 (2.1, 4.4)	2.9 (2.0, 4.3)*	2.8 (2.1, 4.2)	2.0 (1.1, 2.8)*	0.305	0.007

Data are presented as mean ± standard deviation or median (interquartile range)

^a^ Comparisons of hemodynamics between both groups at 0 h; ^b^ Comparisons of hemodynamics between both groups at 6 h; * *P* < 0.05 for comparisons of hemodynamics between 0 h and 6 h within one group

*VAC* ventricular-arterial coupling, *HR* heart rate, *CVP* central venous pressure, *SAP* systolic arterial pressure, *DAP* diastolic arterial pressure, *MAP* mean arterial pressure, *VTI* velocity-time integral, *SV* stroke volume, *LVEDV* left ventricular end-diastolic volume, *LVESV* left ventricular end-systolic volume, *LVEF* left ventricular ejection fraction, *Ea* effective arterial elastance, *Ees* left ventricular end-systolic elastance

## Discussion

In this pilot study, we validated the feasibility of dynamic assessment of the left VAC by TTE to guide the adjustment of treatment during the initial resuscitation in patients with septic shock. The results demonstrated that optimizing the left VAC during the initial resuscitation from septic shock was associated with improved lactate clearance while likely reducing the 28-days mortality and the infused dose of norepinephrine. As EGDT just provides a series of predefined hemodynamic goals and seemly cannot precisely guide subsequent treatment adjustment [27], subsequent adjustment of inotropes and vasoactive agents are typically subjective to the physician’s discretion. Thus, our findings provide a new insight into the management of septic shock that quantifying cardiac contractility, arterial load and the ventricular-arterial interactions in a common integrated framework may make adjustment of inotropes and vasoactive agents more precise and logical during the initial resuscitation of septic shock.

To our knowledge, this is the first study to integrate the approach of optimizing the left VAC into the usual care practice in septic shock patients. Our results suggested that the resuscitation protocol based on optimizing the left VAC significantly reduced the Ea/Ees ratio and improved lactate clearance rate, indicating that optimizing the left VAC was related to improved systemic tissue perfusion. This finding was consistent with the results from a previous study [11]. Of note, compared with the usual care, the improvement of VAC in the VAC-optimized group was primarily caused by a decreased Ea value that resulted from a slightly lower infused dose of NE. It has been demonstrated that NE has extensive effects on the resistive (i.e., total peripheral resistance) and pulsatile component (i.e., arterial compliance) of arterial load and thus alters arterial elastance [18, 28, 29]. Therefore, increased arterial load by a high dose of NE could lead to ventricular-arterial uncoupling. However, ventricular-arterial uncoupling was reported to be associated with the oxygen consumption (VO_2_) responsiveness to oxygen delivery (DO_2_) increases [30], and VO_2_ responsiveness was associated with high lactate [31]. Moreover, a previous study reported that restoring MAP and vascular waterfall with NE may not be systematically associated with improvement of tissue perfusion [29], and several randomized studies [32, 33] did not reveal any clinical benefit with the use of a higher dose of vasopressor to maintain a higher MAP. Thus, under the premise of ensuring a minimum MAP, early weaning of NE may be associated with a well-coupled ventricular-arterial interaction and thus better tissue perfusion.

Additionally, we observed a slightly lower 28-days mortality in the VAC-optimized group. This result is expected because lactate clearance has been extensively described as a predictor of mortality in critical illness and high lactate clearance is associated with a better clinical prognosis [34]. The survival benefit without statistical significance might attribute to the following factors: firstly, the Ea/Ees ratio in the VAC-optimized group was not reduced to the target value; secondly, the Ea/Ees ratio in the usual care group was also optimized after the 6 h of initial resuscitation. Lastly, the small sample size might also contribute to the nonsignificant survival benefit of left VAC optimization. Hence, whether optimizing the left VAC has survival benefit need to be further confirmed in future studies with a larger sample size, in which a more reliable resuscitation algorithm that can accurately optimize the left VAC to the target value should be established.

Applying the concept of VAC in the hemodynamic management of septic shock offers an important advantage that the left VAC facilitates a better understanding of the pathophysiologic changes of hemodynamics during the initial resuscitation. Different from those traditional cardiovascular variables (e.g., LVEF, systemic vascular resistance, arterial compliance) which solely reflects cardiac contractility or arterial load, the left VAC (i.e., the Ea/Ees ratio) comprehensively takes account of both cardiac contractility and arterial load in a single variable and describes the ventricular-arterial system as an interconnected system but not as isolated structures [6]. During the resuscitation of septic shock, the treatment measures exhibit a complex effect on cardiovascular performance [16–18]. For instance, norepinephrine can not only increase arterial pressure by restoring vasomotor tone but also improve cardiac output by improving cardiac contractility and increasing cardiac preload [35]. Thus, the use of traditional cardiovascular variables is difficult to simultaneously track the norepinephrine-induced changes in cardiac function and arterial load. From this perspective, the left VAC can circumvent this limitation because it can quantify both cardiac contractility and arterial load in a common integrated framework and thus represents the interactions between the cardiac and arterial systems.

Several limitations in our study should be recognized. Firstly, our study has some methodological shortcomings. As no information regarding the mortality of patients who received VAC-optimized resuscitation could be referenced previously, the sample size calculation was based on one of our previous studies [26], which reported the mortalities of septic shock patients with and without left ventricular-arterial uncoupling. This sample size calculation is unreasonable. If the calculation is based on the mortality data in this pilot study, a larger sample size is required to obtain the same statistical power. Additionally, the unblinded design in our study could introduce potential bias into the results. Secondly, the resuscitation algorithm based on optimizing the left VAC is not precise enough to guide the treatment adjustment. In our study, the resuscitation algorithm can only guide the direction for when to adjust the inotropes and vasoactive drugs, it fails to tell us how to regulate the infused dose at each adjustment. Practically, it is difficult to accurately adjust the dose of inotropes and vasoactive drugs for reaching the preset target of VAC because each adjustment will have a complex impact on the VAC and thus results in an unexpected comprehensive effect. Therefore, the development of an accurate and reliable VAC optimization-based resuscitation protocol may be an interesting direction for future researches. Thirdly, the selected septic shock patients represent another limitation in our study. We only enrolled those septic shock patients who had an uncoupled ventricular-arterial interaction at baseline. Thus, our findings are inapplicable to the population of patients with ventricular-arterial coupling at the time of septic shock onset. Lastly, the estimated methods of Ea and Ees represent a challenge for the reliability and reproducibility of our results. In our study, the Ees estimation was based on the noninvasive single-beat method [25] in which a load-independent linear ESPVR with a constant volume axis intercept was assumed. However, the volume axis intercept is not always constant and can be changed under varied pathophysiological conditions. For instance, the volume axis intercept was reported to be significantly related to cardiac contractility [36]. Hence, one can expect that sepsis-induced cardiomyopathy might affect the volume axis intercept and the Ees estimation to some extent. In addition, the Ea was estimated as (0.9 × SAP)/SV in our study. However, a previous study suggested that the Ea calculation based on MAP/SV provided a more consistent estimation of Ea with a minimal bias and narrow limits of agreement [37]. Although the use of 90% of SAP, instead of MAP, as a surrogate of left ventricular end-systolic pressure would affect the precision of absolute values of Ea and Ees, it would not affect the precision of Ea/Ees ratio estimation due to the similar influences on Ea and Ees [17].

## Conclusion

A resuscitation strategy based on optimizing the left VAC by adjusting the inotropes and vasoactive drugs is feasible during the initial resuscitation of septic shock. Optimizing the left VAC was associated with improved lactate clearance, while likely having a beneficial effect on the prognosis in patients with septic shock. Given the low methodological quality in this study, our findings should be further confirmed in future larger studies with high methodological quality, and a more reliable resuscitation algorithm that can accurately optimize the left VAC is warranted.

## Supplementary Information

**Additional file 1: Table S1**. Intra-observer reproducibility for transthoracic echocardiography examination. **Table S2**. Dose of norepinephrine and inotropes at each time point. **Table S3**. Changes of hemodynamic variables at each time point.

## Data Availability

The datasets used and/or analyzed during the current study are available from the corresponding author on reasonable request.

## Abbreviations

VAC: Ventricular-arterial coupling
Ea: Effective arterial elastance
Ees: Left ventricular effective end-systolic elastance
ESPVR: End-systolic pressure–volume relationship
SV: Stroke volume
SD: Standard deviation
ICU: Intensive care unit
NE: Norepinephrine
TTE: Transthoracic echocardiography
CVP: Central venous pressure
IMV: Invasive mechanical ventilation
MAP: Mean arterial pressure
LVEDV: Left ventricular end-diastolic volume
LVESV: Left ventricular end-systolic volume
LVEF: Left ventricular ejection fraction
VTI: aortic velocity-time integral
LVOT: Left ventricular outflow tract
T_pre-e_: pre-ejection time
T_tot-s_: total systolic time
HR: heart rate
SAP: Systolic arterial pressure
DAP: Diastolic arterial pressure
CI: Cardiac index
APACHE: Acute physiology and chronic health evaluation
SOFA: Sequential organ failure assessment
PEEP: Positive end-expiratory pressure
IQR: Interquartile range
EGDT: Early goal-directed therapy
VO_2_: oxygen consumption
DO_2_: Oxygen delivery
