# Supplementary material for: Optimizing left ventricular-arterial coupling during the initial resuscitation in septic shock – a pilot prospective randomized study
Source: BMC Anesthesiol. 2022 Jan 21;22:31. doi: 10.1186/s12871-021-01553-w (PMC8781114; doi:10.1186/s12871-021-01553-w)
Supplement: Supplementary file 1 — Additional file 1: Table S1. Intra-observer reproducibility for transthoracic echocardiography examination. Table S2. Dose of norepinephrine and inotropes at each time point. Table S3. Changes of hemodynamic variables at each time point. [file 12871_2021_1553_MOESM1_ESM.docx]

Table S1. Intra-observer reproducibility for transthoracic echocardiography examination

| Variables | LVEDV | LVESV | VTI | T_pre-e_ | T_tot-s_ |
| --- | --- | --- | --- | --- | --- |
| Coefficient of variation (%) | 3.4 (2.6 - 4.3) | 4.3 (3.3 - 5.3) | 1.8 (1.4 - 2.2) | 4.4 (2.8 - 5.9) | 2.3 (1.7 - 2.9) |
| Least significant change (%) | 5.5 (4.1 – 6.9) | 6.9 (5.3 – 8.5) | 2.9 (2.3 – 3.5) | 7.0 (4.5 - 9.5) | 3.7 (2.7 - 4.7) |

Data was presented as mean value and its corresponding 95% CI.

LVEDV left ventricular end-diastolic volume; LVESV left ventricular end-systolic volume; VTI aortic velocity-time integral; T_pre-e_ pre-ejection time; T_tot-s_ total systolic time.

Table S2. Dose of norepinephrine and inotropes at each time point

|  | VAC-optimized group (n=40) | | | | Usual care group (n = 42) | | | |
| --- | --- | --- | --- | --- | --- | --- | --- | --- |
|  | 0h | 2h | 4h | 6h | 0h | 2h | 4h | 6h |
| No. of patients received NE | 40 | 40 | 39 | 30 | 42 | 42 | 42 | 40 |
| *Infused dose (μg/kg/min)* | 0.286 (0.189, 0.415) | 0.208 (0.123, 0.301) ^a^ | 0.164 (0.090, 0.242) ^a^ | 0.177 (0.109, 0.310) ^a^ | 0.213 (0.172, 0.444) | 0.247 (0.177, 0.444) | 0.217 (0.153, 0.433)* | 0.208 (0.126, 0.407) ^a^ |
| No. of patients received dobutamine | 17 | 17 | 17 | 17 | 6 | 10 | 10 | 10 |
| *Infused dose (μg/kg/min)* | 3.87 ± 1.51 | 5.11 ± 2.56 ^a^ | 5.52 ± 2.59 ^a^ | 5.60 ± 2.60 ^a^ | 5.64 ± 1.26* | 6.83 ± 2.93 | 6.64 ± 2.79 | 6.64 ± 2.79 |
| No. of patients received levosimendan | 0 | 1 | 1 | 1 | 0 | 0 | 0 | 0 |
| No. of patients received milrinone (n) | 2 | 2 | 2 | 2 | 0 | 0 | 0 | 0 |

Data are presented as mean ± standard deviation or median (interquartile range).

^a^*P* < 0.05 for comparison between the reference (0h) and each time point within one group; **P* < 0.05 for comparison between the VAC-optimized group and the usual care group at each time point.

VAC ventricular-arterial coupling; NE norepinephrine; IQR interquartile range.

Table S3. Changes of hemodynamic variables at each time point

| Hemodynamic variables | VAC-optimized group (n=40) | | | | Usual care group (n = 42) | | | |
| --- | --- | --- | --- | --- | --- | --- | --- | --- |
|  | 0h | 2h | 4h | 6h | 0h | 2h | 4h | 6h |
| HR (beats/min) | 113 ± 20 | 109 ± 16^a^ | 100 ± 12^a^ | 94 ± 10^a^ | 109 ± 19 | 106 ± 18^a^ | 97 ± 13^a^ | 90 ± 11^a^ |
| CVP (mmHg) | 9 ± 3 | 9 ± 2 | 8 ± 2 | 9 ± 2 | 8 ± 3 | 8 ± 2* | 9 ± 2^a^* | 10 ± 2^a^* |
| SAP (mmHg) | 90 ± 5 | 108 ± 7^a^ | 111 ± 8^a^ | 114 ± 10^a^ | 92 ± 6 | 111 ± 8^a^ | 121 ± 10^a^* | 124 ± 11^a^* |
| DAP (mmHg) | 43 ± 4 | 57 ± 4^a^ | 61 ± 5^a^ | 63 ± 7^a^ | 44 ± 6 | 57 ± 5^a^ | 63 ± 5^a^* | 63 ± 7^a^ |
| MAP (mmHg) | 58 ± 4 | 74 ± 4^a^ | 78 ± 6^a^ | 80 ± 7^a^ | 60 ± 5 | 75 ± 5^a^ | 83 ± 7^a^* | 83 ± 8^a^ |
| VTI (cm) | 15.2 ± 1.6 | 15.7 ± 1.3^a^ | 16.2 ± 1.6^a^ | 16.9 ± 1.5^a^ | 14.9 ± 1.7 | 15.2 ± 1.4^a^ | 15.7 ± 1.3^a^ | 16.2 ± 1.4^a^* |
| SV (mL) | 44 ± 4 | 46 ± 5^a^ | 48 ± 6^a^ | 49 ± 5^a^ | 44 ± 6 | 45 ± 6^a^ | 47 ± 6^a^ | 48 ± 7^a^ |
| LVEDV (mL) | 96 ± 12 | 96 ± 11 | 97 ± 12^a^ | 98 ± 11^a^ | 93 ± 11 | 94 ± 11^a^ | 95 ± 11^a^ | 96 ± 11^a^ |
| LVESV (mL) | 50 ± 9 | 49 ± 8^a^ | 50 ± 8 | 49 ± 7^a^ | 49 ± 7 | 49 ± 6 | 48 ± 6 | 48 ± 5 |
| LVEF (%) | 46 ± 5 | 48 ± 4^a^ | 49 ± 4^a^ | 50 ± 4^a^ | 48 ± 4 | 48 ± 3 | 49 ± 3^a^ | 50 ± 3^a^ |
| Cardiac index (L/min/m^2^) | 3.1 ± 0.6 | 3.1 ± 0.5 | 3.0 ± 0.4^a^ | 2.9 ± 0.4^a^ | 2.9 ± 0.7 | 2.9 ± 0.6 | 2.8 ± 0.5^a^* | 2.6 ± 0.4^a^* |
| Ea (mmHg/mL) | 1.89 ± 0.15 | 2.14 ± 0.25^a^ | 2.15 ± 0.32^a^ | 2.12 ± 0.24^a^ | 1.90 ± 0.19 | 2.21± 0.25^a^ | 2.36 ± 0.30^a^* | 2.35 ± 0.33^a^* |
| Ees (mmHg/mL) | 1.28 ± 0.12 | 1.72 ± 0.24^a^ | 1.85 ± 0.31^a^ | 1.95 ± 0.32^a^ | 1.32 ± 0.14 | 1.66 ± 0.20^a^ | 1.86 ± 0.23^a^ | 1.81 ± 0.26^a^* |
| Ea/Ees ratio | 1.46 ± 0.10 | 1.25 ± 0.11^a^ | 1.17 ± 0.12^a^ | 1.10 ± 0.15^a^ | 1.45 ± 0.16 | 1.33 ± 0.08^a^* | 1.27 ± 0.07^a^* | 1.30 ± 0.11^a^* |

Data are presented as mean ± standard deviation.

^a^*P* < 0.05 for comparison between each time point and the reference (0h) within one group; **P* < 0.05 for comparison between the VAC-optimized group and the usual care group at each time point.

VAC ventricular-arterial coupling; HR heart rate; CVP central venous pressure; SAP systolic arterial pressure; DAP diastolic arterial pressure; MAP mean arterial pressure; VTI velocity-time integral; SV stroke volume; LVEDV left ventricular end-diastolic volume; LVESV left ventricular end-systolic volume; LVEF left ventricular ejection fraction; Ea effective arterial elastance; Ees left ventricular end-systolic elastance.
